# *Chlamydia trachomatis* plasmid-encoding Pgp3 protein induces secretion of distinct inflammatory signatures from HeLa cervical epithelial cells

**DOI:** 10.1186/s12866-023-02802-3

**Published:** 2023-03-04

**Authors:** Heng Choon Cheong, Yi Ying Cheok, Yee Teng Chan, Ting Fang Tang, Sofiah Sulaiman, Chung Yeng Looi, Rishein Gupta, Bernard Arulanandam, Li-Yen Chang, Won Fen Wong

**Affiliations:** 1 Department of Medical Microbiology, Faculty of Medicine, Universiti Malaya, 50603 Kuala Lumpur, Malaysia; 2 Department of Obstetrics and Gynecology, Faculty of Medicine, Universiti Malaya, 50603 Kuala Lumpur, Malaysia; 3 School of Biosciences, Faculty of Health and Medical Sciences, Taylor’s University, 47500 Subang Jaya, Selangor Malaysia; 4 South Texas Center for Emerging Infectious Diseases, University of Texas at San Antonio, San Antonio, TX USA; 5 Department of Immunology, Tufts University School of Medicine, Boston, MA 02111 USA

**Keywords:** *Chlamydia trachomatis*, Pgp3, Cytokine, Chemokine, IL-6, IL-8, TNFAIP, CXCL1

## Abstract

**Background:**

Genital *Chlamydia trachomatis* infection is the most common bacterial sexual transmitted disease that causes severe complications including pelvic inflammatory disease, ectopic pregnancy, and infertility in females. The Pgp3 protein encoded by *C. trachomatis* plasmid has been speculated to be an important player in chlamydial pathogenesis. However, the precise function of this protein is unknown and thus remains to be thoroughly investigated.

**Methods:**

In this study, we synthesized Pgp3 protein for in vitro stimulation in the Hela cervical carcinoma cells.

**Results and conclusion:**

We showed that Pgp3 induced prominent expression of host inflammatory cytokine genes including interleukin-6 (*IL-6*), *IL-8*, tumor necrosis factor alpha-induced protein 3 (*TNFAIP3*), and chemokine C-X-C motif ligand 1 (*CXCL1*), implying a possible role of Pgp3 in modulating the inflammatory reaction in the host.

**Supplementary Information:**

The online version contains supplementary material available at 10.1186/s12866-023-02802-3.

## Introduction

*Chlamydia trachomatis* is an obligate intracellular pathogen that predominantly infects the mucosal epithelial cells lining lower regions of the female reproductive tract. Genital chlamydial infection represents an important public health concern and is a significant cause of morbidity across the globe with 131 million cases being reported annually [1]. Various adverse obstetrics and gynecological outcomes are associated with chlamydial infection such as preterm delivery, premature rupture of membranes, and spontaneous abortion [2–5]. Although it is widely believed that chlamydial diseases are the results of chronic and overt inflammation in response to chlamydial infection, the precise mechanism whereby these immune responses are produced, and the specific chlamydial antigen that initiates the destructive aspect of such reaction remain poorly understood [6].

Almost all characterized strains of *C. trachomatis* naturally carry a conserved 7.5 kbp plasmid [7]. A total of eight open reading frames (ORFs) have been identified in the chlamydial plasmid, each (*Pgp1–8*) is expressed during infection [8]. The *Pgp3* gene specifies a 28 kDa protein that is produced as a stable trimer during infection. As opposed to the other seven plasmid proteins that are localized strictly within the chlamydial inclusions, Pgp3 is secreted into the host cell cytosol or remain in association with the chlamydial outer membrane complex (COMC) during infection [9, 10]. Pgp3 is highly immunogenic, and most patients infected with *C. trachomatis* develop antibodies to the protein, suggesting its possible involvement in the development of host immunopathology [11–14].

In this present study, we examined the cellular responses following Pgp3 stimulation. Our results showed that Pgp3 induced prominent expression of several genes encoding pro-inflammatory cytokines including interleukin 6 (*IL-6*), interleukin 8 (*IL-8*), and C-X-C motif chemokine ligand 1 (*CXCL1*). Tumor necrosis factor, alpha-induced protein 3 (*TNFAIP3*), a gene with a known role in immunosuppression was also upregulated, suggesting a role of Pgp3 in the modulation of host immune responses.

## Materials and methods

### *C. trachomatis* infection

Hela-229 cells (ATCC CCL-2.1) were infected with *C. trachomatis* Serovar D (ATCC VR-885) using a method previously described with minor modifications [15]. Briefly, confluent monolayer cells were washed with Hank’s Balanced Salt Solution (HBSS) and then treated with Diethylaminoethyl (DEAE)-dextran (30 μg/mL) in HBSS for 35 min prior to infection. DEAE-dextran treated cells were infected with *C. trachomatis* resuspended in sucrose phosphate glutamate buffer (SPG), followed by incubation in a shaking incubator with constant shaking for 2 hr. After that, Dulbecco’s Modified Eagle Medium (DMEM) supplemented with 10% fetal bovine serum (FBS), 10 μg/mL gentamicin, and 1 μg/mL cycloheximide was added to the flask and the cells were incubated at 37 °C, 5% CO_2_ for 72 hr.

### Pgp3 cloning

*C. trachomatis* DNA was extracted based on procedures outlined previously with modifications [16]. Cells at 72 h post-infection were detached mechanically from the tissue culture flask using 5 mm borosilicate glass beads. The detached cells were further disrupted in a 50 mL centrifuge tube and the resultant lysed cells were centrifuged at 500×*g*, 4 °C for 10 min to remove cell debris. Then, the supernatant was centrifuged in a high-speed centrifuge at 14, 000×*g*, 4 °C for 90 min. The supernatant was discarded and the pelleted elementary body (EB) was resuspended in 180 μL buffer ATL for DNA extraction using the DNeasy Blood & Tissue kit (Qiagen, Hilden, Germany). In brief, the resuspended EBs were first mixed with 20 μL Proteinase K and then incubated in a dry block heater at 56 °C for 3 hr. After adding 200 μL Buffer AL and 200 μL 100% ethanol, the mixture was centrifuged through a DNeasy Mini spin column. The spin column was washed, and DNA was eluted with 100 μL Buffer AE and then kept at − 20 °C prior until further processing.

Chlamydial *Pgp3* gene was obtained from the extracted *C. trachomatis* DNA using gene-specific primer pairs as described previously with modifications to the restriction sites as outlined in Table 1 [9]. Amplification was performed using Q5 high-fidelity DNA polymerase (New England Biolabs, MA, USA) using the following cycling profile: 95 °C for 5 min, 35 cycles of 95 °C for 30 sec, and 60 °C for 45 sec. The amplicons were ligated into the vector pTriEx-3 Hygro (Novagen, NJ, USA) with 8× histidine residues fused to the C-terminus and subsequently transformed into the *Escherichia coli* cloning host NovaBlue (Novagen). After sequence verification of positive recombinant clones, the recombinant plasmids were then transformed into the *E. coli* expression host RosettaBlue (DE3) pLacI (Novagen).

**Table 1:** Cloning primers of Pgp3 gene and primers used for qRT-PCR analysis of immune-related genes

Target gene	Forward primer (5′-3′)	Reverse primer (5′-3′)	Amplicon size (bp)
*Pgp3*	CATG*CCATGG*GAAATTCTGGTTTTTATTTG	CGTA/CTCGAG/AGCGTTTGTTGAGGT	792
*CXCL1*	GCGGAAAGCTTGCCTCAA	TCAGCATCTTTTCGATGATTTTCTT	62
*GM-CSF*	CACTGCTGCTGAGATGAATGAAA	GTCTGTAGGCAGGTCGGCTC	78
*IL-1α*	TGTATGTGACTGCCCAAGATG	TTAGTGCCGTGAGTTTCCC	121
*IL-5*	GAGACCTTGGCACTGCTTTC	CAGTACCCCCTTGCACAGTT	157
*IL-6*	CCACTCACCTCTTCAGAACG	CATCTTTGGAAGGTTCAGGTTG	150
*IL-8*	CCTGATTTCTGCAGCTCTGT	TCTGCACCCAGTTTTCCTTG	221
*TNF-α*	ACTTTGGAGTGATCGGCC	GCTTGAGGGTTTGCTACAAC	139
*TLR2*	CAGGTGACTGCTCGGAGTTC	CACAACTACCAGTTGAAAGCAGTGA	171

Restriction sites: * *, *Nco*I site; / /, *Xho*I site

### Protein synthesis and purification

Transformed *E. coli* expression host was cultured in selective lysogeny broth containing 50 μg/mL ampicillin, 12.5 μg/mL tetracycline, and 34 μg/mL chloramphenicol supplemented with 1% glucose. Cells were grown at 37 °C with aeration at 250 rpm to an OD_600_ of ≥0.7, upon which expression was induced with 1 mM isopropyl β-D-1-thiogalactopyranoside (IPTG). After 4 hr, the bacterial pellets were obtained by centrifugation at 10, 000×*g* for 10 min at 4 °C. The pelleted cells were resuspended in 10 mL lysis buffer (10 mM Na_2_HPO_4_, 10 mM NaH_2_PO_4_, 500 mM NaCl, 2 mg/mL lysozyme, pH 7.4), incubated on ice for 30 min, and then sonicated in a Branson Sonifier 250. The resultant crude lysate was centrifuged at 10, 000×*g* for 10 min at 4 °C and the clarified supernatant containing the soluble proteins was then collected for purification.

Pgp3 protein was purified by immobilized metal affinity chromatography (IMAC) using HisTrap HP column (GE Healthcare, UK) on an AKTA Purifier System (GE Healthcare, UK). Purification of Pgp3 was performed natively by applying the clarified supernatant containing the soluble Pgp3 onto the column pre-equilibrated with binding buffer (10 mM Na_2_HPO_4_, 10 mM NaH_2_PO_4_, 500 mM NaCl, and 20 mM imidazole). Unspecific proteins were removed by washing the column with 5 column volumes of binding buffer and the protein of interest was eluted in elution buffer (10 mM Na_2_HPO_4_, 10 mM NaH_2_PO_4_, 500 mM NaCl, and 500 mM imidazole). The eluted proteins were collected for immunoblot analyses. Eluted Pgp3 fractions desalted using HiTrap desalting column (GE Healthcare, UK) fitted onto an AKTA purifier system (GE Healthcare, UK). Desalted Pgp3 was depyrogenated with Pierce high-capacity endotoxin removal spin column (Thermo Fisher Scientific, MA, USA) in accordance with the manufacturer’s protocols. Depyrogenated Pgp3 was further concentrated using Vivaspin 6 centrifugal concentrator (Sartorius AG, Germany). Protein concentration was determined using Pierce BCA protein assay kit (Thermo Fisher Scientific, MA, USA). The concentration of endotoxins present in the depyrogenated Pgp3 was assayed using HEK-Blue lipopolysaccharide (LPS) detection kit 2 (Invivogen, CA, USA). In brief, HEK-Blue cells were added to each well to a concentration of 4 × 10^5^ cells/mL and subsequently incubated overnight at 37 °C in a humified incubator at 5% CO_2_ atmosphere. Following overnight incubation, 20 μL of cell supernatants from each standard and unknown were mixed 180 μL of QUANTI-Blue reagent (Invivogen, CA, USA) and incubated for 6 hr at 37 °C. Absorbance at 620 nm was read with a Synergy HTX microplate reader (BioTek Instruments, VT, USA).

### Gel electrophoresis and immunoblotting

Purified Pgp3 was separated with sodium dodecyl sulfate–polyacrylamide gel electrophoresis (SDS-PAGE) and then stained with Coomassie brilliant blue G-250 (Bio-Rad, CA, USA). Native-PAGE was performed with the same procedures as SDS-PAGE, except without heat denaturing and treatments with reducing agents including SDS and β-mercaptoethanol in the experiments. For immunoblot analysis, the separated proteins were blotted onto polyvinylidene difluoride (PVDF) membrane and then blocked with 5% bovine serum albumin (Merck Millipore, MA, USA). Detection of the His-tagged recombinant Pgp3 was carried out by incubating the membrane with HisDectector nickel alkaline phosphatase (AP)-conjugate (KPL, MD, USA) diluted 1: 5000 for 1 hr at room temperature. Subsequently, the color was developed by adding NBT-BCIP substrate (Promega, WI, USA).

### Peptide mass fingerprinting

Purified Pgp3 was resolved using SDS-PAGE and then stained overnight with colloidal Coomassie blue G-250 (Bio-Rad, CA, USA). Protein band corresponding to the molecular weight of Pgp3 monomer (~ 28 kDa) was excised from stained gel. The band was destained, dried, and then digested with trypsin for 2 hr at 37 °C. Digested gel plugs were solvent extracted twice using 0.1% trifluoroacetic acid (Sigma-Aldrich, MO, USA) in 50% acetonitrile for 30 min at room temperature and transferred to new wells. Following overnight drying at 37 °C, the dried peptides were reconstituted with 10 μL of 0.1% formic acid (Sigma-Aldrich, MO, USA). The tryptic peptides were desalted by using ZipTip with 0.1% formic acid and eluted with 0.1% formic acid in 50% acetonitrile. Eluted peptides were mixed with equal volume of matrix solution (6 mg/mL a-cyano-4-hydroxycinnamic acid in 70% acetonitrile, 0.1% formic acid) and spotted onto the sample slide before analyzed on a 5800 Plus Matrix-assisted laser desorption/ionization-tandem time of flight (MALDI TOF/TOF) analyzer (Applied Biosystems/SCIEX, CA, USA). Protein identity was confirmed by searching the Swiss-Prot database using the MASCOT search engine with the following parameters: enzyme – trypsin, missed cleavage – 1, variable modification – 2; (I) carbamidomethylation of cysteine residues, and (II) allowed variable modifications of methionine oxidation, MS precursor ion mass tolerance – 100 ppm, tandem mass spectrometry (MS/MS) fragment ion mass tolerance − 0.2 Da, and mass values restricted to monoisotopic, as previously described [17].

### Cell stimulation

A day prior to stimulation, Hela-229 cells were seeded at a density of 1 × 10^5^ cells/mL in DMEM supplemented with 10% FBS in a 6-well plate and grown overnight at 37 °C, 5% CO_2_. The serum containing DMEM was removed, washed once with DPBS, before replacing the medium with serum-free DMEM. After that, cells were treated with Pgp3 at final concentrations of 5 μg/mL and 10 μg/mL. Cells stimulated with DPBS and 0.1 μg/mL LPS were prepared alongside to serve as a negative and positive control, respectively. The cells were incubated for 24 h at 37 °C, 5% CO_2_ before harvested for extraction of RNA.

### RNA extraction

RNA was extracted from HeLa-229 cells using Trizol reagent (Invitrogen, CA, USA). A total of 1 mL Trizol reagent and 200 μL chloroform was added into cell pellet, vortexed and left for 3 min at room temperature before centrifugation at 12,000×*g* for 15 min at 4 °C. Then, the upper layer of the sample mixture was collected and transferred to a tube containing 400 μL of isopropanol. Sample was then mixed and left for 10 min at room temperature before centrifugation. RNA pellet was washed using 75% ethanol, dissolved in 30 μL of RNase-free water and stored at − 80 °C until further usage.

### Quantitative real-time polymerase chain reaction (qRT-PCR)

Extracted RNAs were converted to cDNA by using iScript cDNA synthesis kit (Bio-Rad, CA, USA) according to the manufacture’s guidelines. qRT-PCR was performed using Kapa SYBR Fast qPCR master mix (2×) kit (KapaBiosystems, MA, USA). The qRT-PCR master mix comprised 5 μL of Kapa SYBR Fast qPCR master mix (2×), 0.2 μL of each 10 μM forward and reverse primers, 0.2 μL of 50× ROX low, 1 μL of template DNA, and 3.4 μL of water. qRT-PCR was performed using the following cycling parameters: 95 °C for 3 min, 40 cycles of 95 °C for 3 seconds, 60 °C for 20 seconds, and a dissociation curve analysis step consisting of 1 cycle of 95 °C for 3 min, 95 °C for 3 seconds, and 60 °C for 20 seconds. The relative fold change of each gene was calculated using the 2^–ΔΔC^_T_ method [18]. All reactions were performed in triplicates and the results were expressed as mean ± standard deviation (SD). The primer sets used in qRT-PCR are listed in Table 1.

### NanoString nCounter analysis

Hybridization of mRNA with both reporter codes and capture probes was carried out as specified by the manufacturer (NanoString Technologies Inc., WA, USA). Following 16 hr incubation at 65 °C on a Veriti thermal cycler (ThermoFisher Scientific, MA, USA), the reaction mix containing 20 ng/μL of RNA, reporter codes, as well as capture probes were transferred to nCounter Prep Station (NanoString Technologies) for processing. The experiment was performed in triplicates from three independent experiments. Gene expressions were analyzed on an nCounter MAX Analysis System using the nCounter PanCancer Pathways Panel for human (NanoString Technologies).

### Expression data and statistical analysis

Data normalization and analysis of gene expression were performed with nSolver analysis software (version 4.0, NanoString Technologies) using the advanced analysis module to calculate the differential expression fold change between samples. Genes with fewer than 30 reads were removed from all samples following normalization of raw data to internal spike-in negative and positive controls as well as reference genes. Heatmap was generated using Morpheus (https://software.broadinstitute.org/morpheus). Z-scores were computed from log_2_-adjusted expression data, following which unsupervised hierarchical clustering of genes was performed by using one minus Pearson’s correlation matrix with average linkage method in Morpheus. The scale of the expression data is based on the maximum and minimum values of each row. Statistical significance for the NanoString data was conducted using the R-statistics by either log-linear regression or simplified negative binomial model, with *P*-values adjusted according to Benjamini-Hochberg method. Volcano plot was generated using GraphPad Prism 9.0 (GraphPad Software, San Diego, CA, USA) Data were analyzed with unpaired two–tailed Student’s *t*–test using GraphPad Prism 9.0 (GraphPad Software) when comparing between two groups. Statistical significance was established when *P* < 0.05*, *P* < 0.01**, *P* < 0.001***, and *P* < 0.0001****.

## Results

### Expression and purification of chlamydial Pgp3 protein

**Fig. 1:**
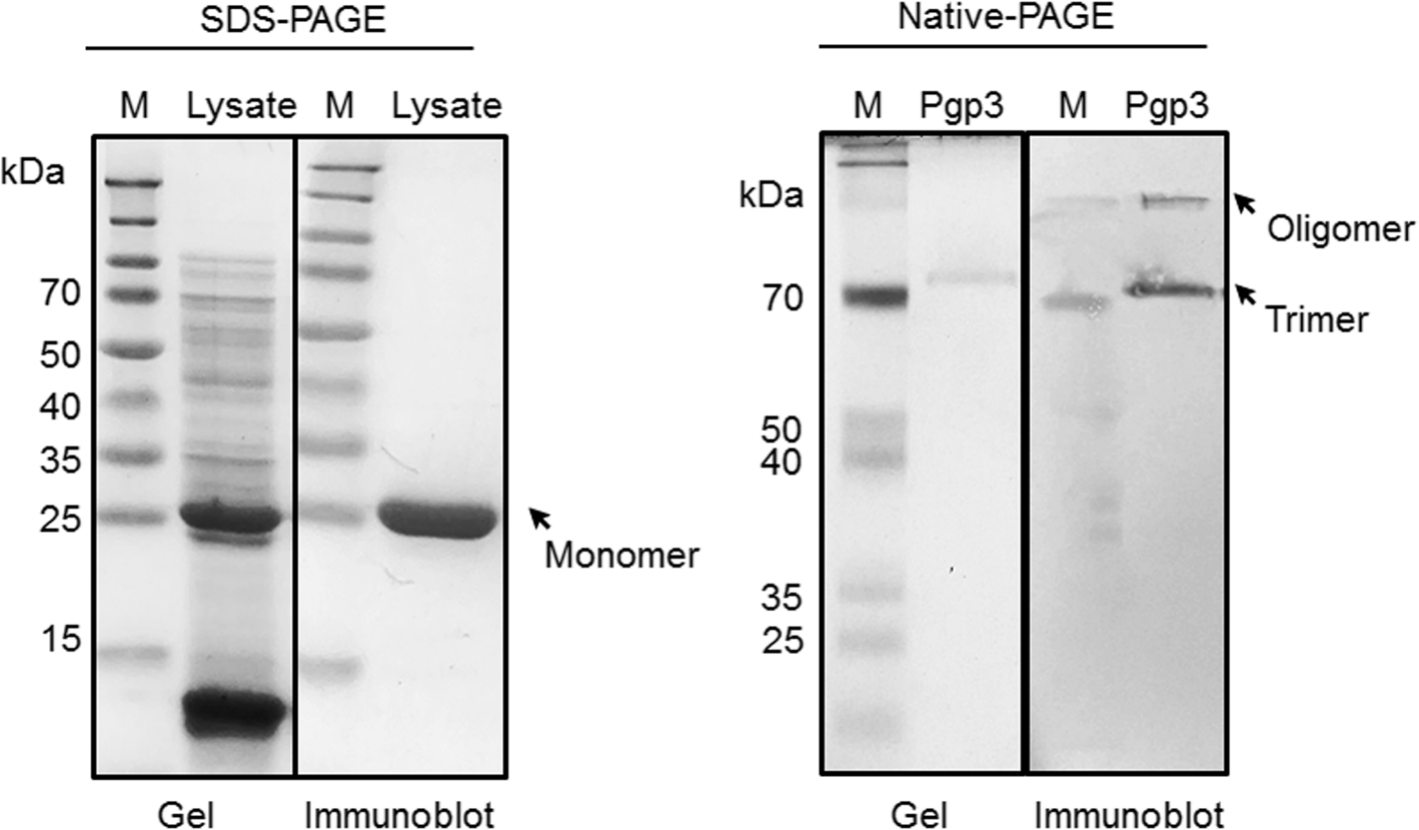
Pgp3 protein synthesis and purification. Left panel: SDS-PAGE gel and immunoblot images of the crude cell lysates derived from Pgp3 transfected cells. Right panel: Native-PAGE gel and immunoblot images of the purified soluble Pgp3 protein fraction. His-conjugated protein was detected using HisDectector nickel alkaline phosphatase (AP)-conjugate. M: Protein marker. Arrows show the position of protein band of the expected molecular weight of Pgp3 monomer (~ 29 kDa), trimer (~ 80 kDa) and oligomer (> 100 kDa)

The *C. trachomatis* Pgp3 protein was synthesized in an *E. coli* expression system and purified from the solution fraction of the cell lysates using immobilized metal affinity chromatography. Expression of recombinant Pgp3 was confirmed by the presence of a protein of approximately 29 kDa bands in SDS-PAGE and immunoblot analyses (Fig. 1, left panel). Majority of the contaminating *E. coli* proteins were removed after purification. When purified product was analyzed on native-PAGE gel, trimer and oligomer bands of Pgp3 can be detected, suggesting native structure of the functional Pgp3 (Fig. 1, right panel).

The identity of the *C. trachomatis* Pgp3 protein was verified by peptide mass fingerprinting using mass spectrometry (Table 2), and was subsequently concentrated and depyrogenated. The endotoxin level of purified Pgp3 was < 1.0 EU/μg.

**Table 2:** Pgp3 identification by MALDI-TOF peptide mass fingerprinting

	Approx. band size (kDa)	Mowse score	Sequence coverage (%)	No. of matched peptides	Calculated mass	Expect value
Pgp3	29.1	89	10	2	28,076	0.0064

### Transcriptome alterations in chlamydial Pgp3-stimulated HeLa cells

**Fig. 2:**
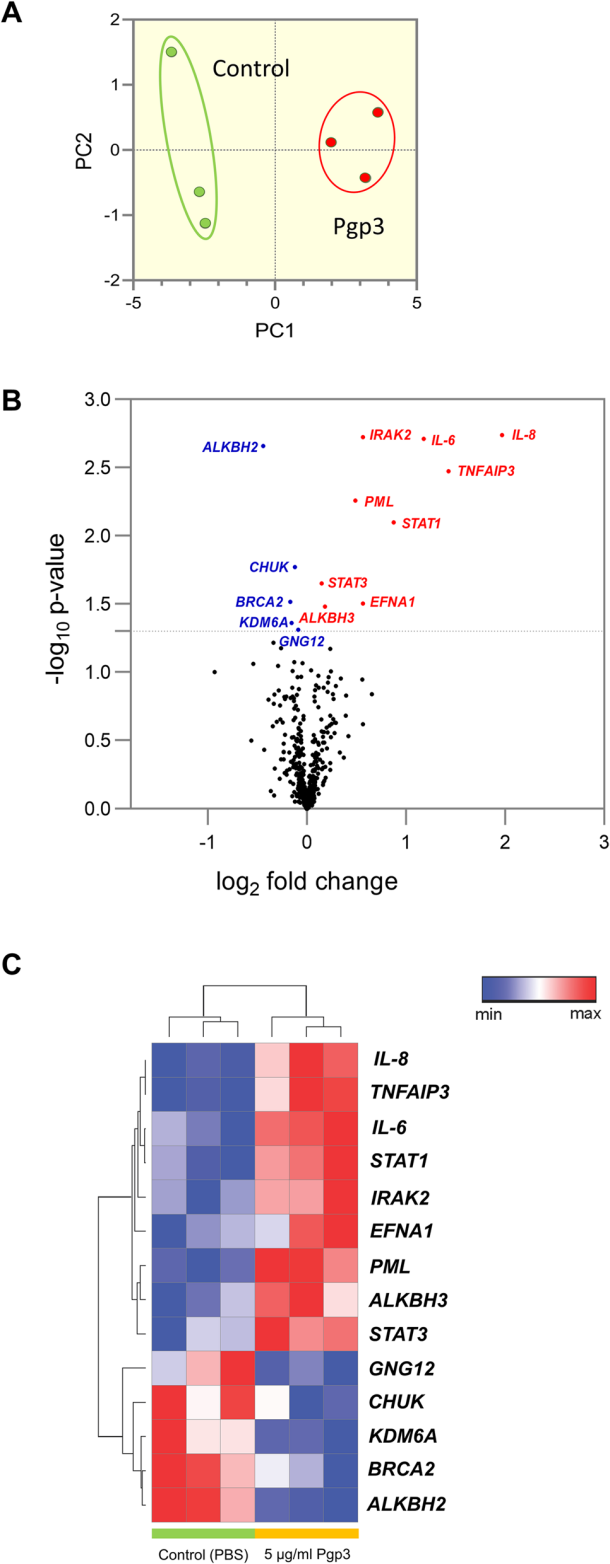
NanoString analysis of mRNA expression in Pgp3-treated epithelial cells. **A** Principal component analysis of the NanoString nCounter mRNA expression in epithelial cells following treatment with 5 μg/mL Pgp3 and PBS. **B** Volcano plot dis-playing differential gene expression caused by Pgp3 between two groups; red: upregulation; blue: downregulation; black: non-significant. **C** A heatmap illustrating unsupervised clustering of statistically significant differentially regulated genes be-tween control (PBS) and 5 μg/mL Pgp3-stimulated epithelial cells; red: upregulation; blue: downregulation

To investigate a broad transcriptomic modification following chlamydial Pgp3 stimulation, cellular total mRNA transcripts in HeLa human cervical epithelial cells were profiled using NanoString nCounter analysis with the nCounter PanCancer Pathways Panel (human codeset) that contains 770 genes with relevant roles in cancer and inflammation. Principal component analysis (PCA) showed a clear separation of clusters according to different treatment groups, indicating distinct mRNA expression profiles of the unstimulated control versus Pgp3-stimulated HeLa cells (Fig. 2A). Overall, a total of 14 significant genes were differentially expressed in the cells exposed to Pgp3 in comparison to untreated controls (Fig. 2B, C). Among the top upregulated genes were those encoding for proinflammatory cytokines, namely *IL8* and *IL6*, which were significantly increased by 1.97- (*P* = 0.00183) and 1.18-fold (*P* = 0.00194) respectively, following treatment with Pgp3 proteins (Fig. 2B, C).

Additionally, Pgp3 protein also induced significant upregulation of tumor necrosis factor α-induced protein 3 (*TNFAIP3*) to 1.46-fold (*P* = 0.00335). *TNFAIP3* encodes for the ubiquitin-modifying enzyme A20, which plays a role in immune suppression through inhibiting the tumor necrosis factor (TNF)-induced signaling pathways upstream of transcription factor NF-κB and TNF-mediated apoptosis [19, 20]. Although *TNFAIP3* functions to limit inflammation, our finding of chlamydial plasmid-encoded Pgp3 protein augmented *TNFAIP3* agrees with a previous report which infected HeLa cells using plasmid-bearing *C. trachomatis* [21]. This suggests a likelihood of Pgp3 in limiting host immune response during chlamydial infection.

Signal transduction and activator of transcription 1 (*STAT1*), on the other hand, was also increased moderately at 0.88-fold (*P* = 0.00797) in response to Pgp3 stimulation (Fig. 2B, C), upregulation of which has previously been found to be crucial for the host anti-chlamydial response [22]. Other genes that showed marginal elevation after Pgp3-treatment include interleukin-1 receptor-associated kinase 2 (*IRAK2*), promyelocytic leukemia (*PML*), ephrin A1 (*EFNA1*), Alkb homolog 3, alpha-ketoglutarate dependent dioxygenase (*ALKBH3*), as well as *STAT3*, at 0.56- (*P* = 0.00189), 0.56- (*P* = 0.0055), 0.49- (*P* = 0.0313), 0.18- (*P* = 0.0331), and 0.15-fold (*P* = 0.0223) respectively.

Several other significant genes were downregulated modestly in the presence of Pgp3 protein, including Alkb homolog 2, alpha-ketoglutarate dependent dioxygenase (*ALKBH2*), breast cancer 2 (*BRCA2*), lysine-specific demethylase 6A (*KDM6A*), conserved helix-loop-helix ubiquitous kinase (*CHUK*), G protein subunit gamma 12 (*GNG12*), which reduced by 0.442- (*P* = 0.0022), 0.17- (*P* = 0.0305), 0.15- (*P* = 0.0436), 0.15- (*P* = 0.0169), and 0.09-fold (*P* = 0.049) respectively (Fig. 2B, C).

### Upregulated expression of IL-6 and IL-8 genes following Pgp3 stimulation

**Fig. 3:**
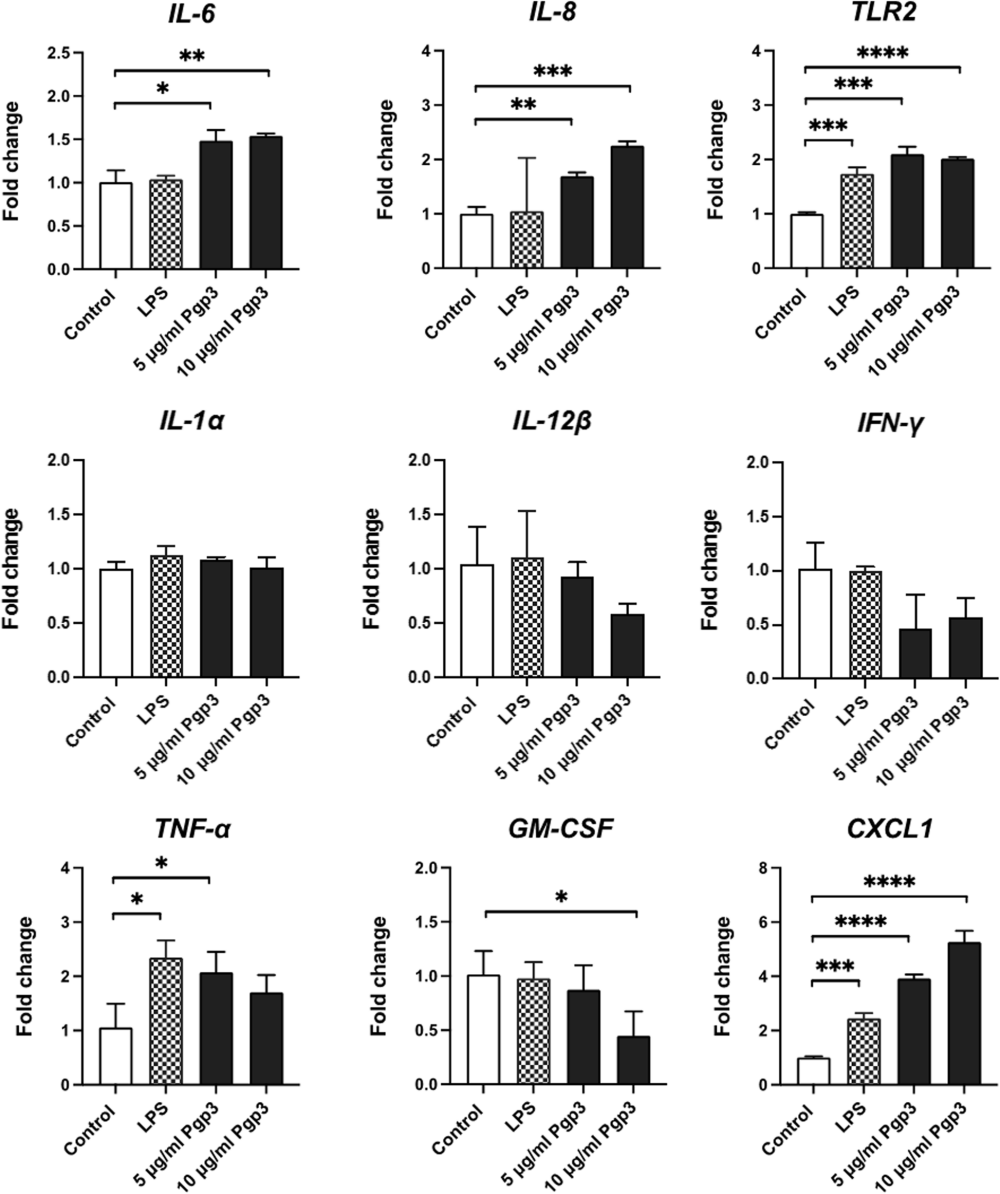
qRT-PCR analysis of immune-related genes. The results were expressed as mean ± SD of an experiment in triplicate. The results are representative of two independent experiments where qRT-PCR was performed in triplicate for each sample. Asterisks denote statistical significance by Student’s t-test when comparison was made against control. *P* < 0.05*, *P* < 0.01**, *P* < 0.001***, and *P* < 0.0001****

To verify the Nanostring data, mRNA transcripts were investigated using qRT-PCR (Fig. 3). Two of the top upregulated genes i.e. IL-6 and IL-8 were examined along with few other important cytokines. Expressions of both IL-6 and IL-8 were increased significantly in a concentration-dependent manner in response to Pgp3 stimulation. IL-8 was upregulated 0.69-fold (*P* < 0.01**) when stimulated with 5 μg/mL of Pgp3, while 10 μg/mL of Pgp3 stimulation caused a 1.25-fold (*P* < 0.001***) increase in IL-8 relative to control. Pgp3 stimulations induced 0.48-fold (*P* < 0.05*) and 0.53-fold (*P* < 0.01**) increases in IL-6 when compared to unstimulated control.

TLR2 activates signaling pathway which triggers *IL-8* gene transactivation [23]. To examine the involvement of TLR2, we have also examined its expression. The transcript levels of *TLR2* were elevated by 1.1-fold (*P* < 0.001***) and 1.01-fold (*P* < 0.0001****) upon exposure to 5 μg/mL and 10 μg/mL Pgp3, respectively. This likely suggests that *IL-8* is activated following TLR2 signal activation by Pgp3 (Fig. 3).

No such substantial increase pattern in a dose-dependent manner was detected in other cytokines examined. Expression of *IL-1α, IL-12β* and *IFN-γ* showed no significant changes following Pgp3 stimulation. *TNF-α* level, on the other hand, was 1.02-fold (*P* < 0.05*) higher when epithelial cells were treated with 5 μg/mL of Pgp3. However, stimulation of epithelial cells with 10 μg/mL of Pgp3, induced an insignificant increase in *TNF-α* transcripts.

Previous study reported that infection by *C. trachomatis* in epithelial cells triggers release a range of cytokines that include IL-1α, granulocyte-macrophage colony-stimulating factor, (GM-CSF), IL-6, as well as IL-8 [24]. Interestingly, chlamydial Pgp3 protein alone could elicit immune response that results in release of IL-6 and IL-8; but not sufficient to activate IL-1α, GM-CSF and other cytokines examined (Fig. 3). Instead, treatment with high concentration (10 μg/mL) of Pgp3 caused a significant downregulation of *GM-CSF*, with a 0.57-fold (*P* < 0.05*) decrease in expression versus control. This signifies a specific route of Pgp3 recognition and signaling pathway activation by epithelial cells.

Other than cytokines, *C. trachomatis* infection is known to induce CXCL1 [24]. Similar to previous study, we showed that the expression of *CXCL1* was also increased significantly in a concentration-dependent manner in response to Pgp3 stimulation (Fig. 3). *CXCL1* mRNA transcripts were elevated by 2.92-fold (*P* < 0.0001****) and 4.26-fold (*P* < 0.0001****) compared to control following stimulations with 5 μg/mL and 10 μg/mL of Pgp3, respectively.

## Discussions

In spite of the differences in tissue tropism, several members of the genus *Chlamydia* namely *C. trachomatis, C. muridarum, C. psittaci, C. suis, C. caviae, C. felis, C. pecorum*, along with animal isolates of *C. pneumoniae*, naturally bear a conserved ~ 7.5 kb plasmid [25–28]. The plasmid is strikingly conserved within *C. trachomatis*, with only ~ 1–3% nucleotide variation between different strains [29, 30]. Although several isolates of *C. trachomatis* devoid of the plasmid have been identified and characterized, they are considered a rare occurrence, suggesting the presence of strong selective pressure favoring the persistence of plasmid in the environment [31–33]. Interestingly, Pgp3 represents the only one of the seven plasmid proteins that is secreted into the host cell during infection [9]. It is therefore possible that Pgp3 may elicit immune responses from host cells and contribute to the induction of host immunopathology.

Although transcriptomic analyses in the present study did not reveal dramatic differences between the Pgp3-exposed cells and control, our data herein showed that stimulation of epithelial cells with Pgp3 increased the levels of expression of *CXCL1* and *IL-8* in a concentration-dependent manner. In addition, the levels of *IL-6* were increased prominently. Epithelial cells are known to produce various proinflammatory cytokines and chemokines that include CXCL1, IL-6, IL-8, as well as GM-CSF following infection with *C. trachomatis* [21, 24, 34]. IL-6, IL-8, CXCL1 are known to promote neutrophil recruitment during inflammation. However, previous reports suggested that neutrophils were insufficient for clearing chlamydial infection. Importantly, both lL-8 and CXCL1, are capable of delaying apoptosis of neutrophils. Therefore, the retention of neutrophils at the site of infection could lead to the development chronic inflammation which represents a hallmark of chlamydial infection [35–37]. This may be further magnified by other immune cells such as monocytes, which have been shown to elevate the production of IL-6 and IL-8 in a co-culture model of chlamydial infection of Hela and THP-1 cells [38]. Interestingly, infection with plasmid-bearing *C. muridarum* attenuated neutrophil apoptosis in mice, which translated to significantly higher levels of cytokines and increased infiltration of mediator of tissue pathology in comparison with the strain lacking the plasmid [39]. Additionally, loss of the *Pgp3* gene in *C. trachomatis* L2 and *C. muridarum* was correlated with both attenuated infectivity and inflammatory infiltration in murine model of genital tract infection [40, 41]. Such attenuated phenotype was also observed in chlamydial infection using a macaque model of ocular infection [42]. These findings collectively suggest that the chlamydial plasmid serves as a contributor to the host inflammatory response to infection.

Toll-like receptors (TLRs) are components of the innate immune system that recognize microbial derived or endogenous ligands. The recognition of these molecules by TLRs trigger the activation of a cascade of downstream events resulting in inflammatory responses [43, 44]. TLR2 is involved in the host immune defense mechanism towards chlamydial infection and may have a potential role in the induction of chronic inflammatory sequelae as oviduct pathology was attenuated in mice knockout for *TLR2* [45–47]. In vivo studies with *C. trachomatis* and *C. muridarum* have revealed a dependency of the chlamydial plasmid for TLR2 activation. Plasmid loss was accompanied by both lowered in vitro as well as in vivo infectivity and reduced pathology in animals [21, 39, 42, 48–53]. Pgp3 was previously shown to elicit the production of a number of proinflammatory cytokines, such as TNF-α, IL-1β, and IL-8 from both mouse Raw 264.7 macrophages and human cell line THP-1. The latter was believed to be dependent on TLR2, activation of which triggers the release of cytokines through the MAPK and (ERK)/MAPK pathways [9, 54]. The data from the current study support these prior findings, whereby parallel increases of both *IL-6* and *IL-8* transcripts were observed with elevation of *TLR2* was observed in response to Pgp3 stimulation. This suggests that Pgp3 is capable of inducing the production of proinflammatory mediators from epithelial cells possibly by activating TLR2, which could contribute to inflammatory injury.

In addition to *IL-6*, *IL-8*, and *CXCL1*, we observed that Pgp3 caused significant upregulation of *TNFAIP3* in epithelial cells. *TNFAIP3* codes for the ubiquitin-modifying enzyme A20 that possesses an anti-inflammatory function by suppressing the activation of NF-κB [19, 20]. This agrees with Porcella et al. whose team found that plasmid-bearing *C. trachomatis* infection upregulated *TNFAIP3* more significantly compared to its plasmidless counterpart. Our result provides additional evidence to incriminate Pgp3 in the immune evasion during chlamydial infection. It has been demonstrated previously that Pgp3 is able to bind to and forms complex with the human LL-37 peptide, effectively neutralizes the peptide in the process [55]. Produced by both epithelial cells and immune cells such as macrophages and neutrophils, the LL-37 is the sole member of the human cathelicidin protein family and is known to have antimicrobial property as well as proinflammatory activity [56]. The binding of LL-37 by Pgp3, therefore, may be a strategy employed by *C. trachomatis* to mitigate the host defense mechanism in the genital tract environment. Recently, Hou et al. showed that Pgp3-LL-37 complex abolished the proinflammatory response in vaginal epithelial cells. Intriguingly, the authors found that while Pgp3 alone was unable to trigger the release of IL-6 and IL-8, the binding of Pgp3 with LL-37 increased the release of IL-6 and IL-8 from neutrophils, which may culminate in the onset of inflammatory injury. A likely scenario that emerged from these observations is that Pgp3 dampens the host inflammatory reaction upon release at the site of infection by binding to LL-37 during chlamydial infection. The contact of Pgp3-LL-37 complex with the arriving neutrophils would probably drive the development of inflammatory process [57]. Our results indicated that epithelial cells responded to Pgp3 treatment, which partially contrast with the findings of the previous study. Differences in cell lines used may partially account for this discrepancy. Future studies involving cells and tissues that are more representative of the biology of the natural host of *C. trachomatis*, such as primary cells and organoids, may help to resolve this disparity in results.

In our previous study involving stimulation of peripheral blood mononuclear cells (PBMC) with the chlamydial protease-like activity factor (CPAF), chlamydial heat shock protein 60 (cHSP60), and the major outer membrane (MOMP) of *C. trachomatis*, there was a strong release of proinflammatory cytokines that includes Tumor necrosis factor alpha (TNFα), IL1β, and IL6. Noteworthy, PBMC derived from patients infected with plasmid-bearing *C. trachomatis* caused a more robust cytokine response [58]. CPAF and cHSP60 are both known to be potent immunogens in humans [59, 60]. Further, cHSP60 has been shown to induce IL-6 production in a variety of cell types including human endothelial cells, smooth muscle cells, as well as macrophages [61]. Importantly, numerous studies have shown an association between the development of severe reproductive sequelae and the production of antibodies to CPAF and cHSP60 [61–65]. Conceivably, the extracellular release of these chlamydial antigens, including Pgp3, may favor the creation of a proinflammatory environment, leading to the onset of immune-mediated pathology that is associated with chlamydial infection.

## Conclusion

In short, the present investigation demonstrated that Pgp3 plays a likely role in the induction of host inflammatory response during *C. trachomatis* and presented evidence for its possible involvement in the evasion of host immune responses. However, the molecular mechanisms as well as the implication of the release of these cytokines, were not addressed. Further investigation of the molecular pathways and extrapolating the data to an animal model of infection, should be performed. This will prove valuable for decoding the additional functions of Pgp3 protein in order to gain a better insight into the role of the plasmid protein in the pathogenesis of *C. trachomatis*, particularly in the context of chlamydial infection-mediated host immunopathology.

## Supplementary Information

**Additional file 1.**

## Data Availability

The datasets used and/or analysed during the current study are available from the corresponding author on reasonable request.

## Abbreviations

ALKBH3: Alkb homolog 3, alpha-ketoglutarate dependent dioxygenase
BRCA2: Breast cancer 2
cHSP60: Chlamydial heat shock protein 60
CHUK: Conserved helix-loop-helix ubiquitous kinase
COMC: Chlamydial outer membrane complex
CPAF: Chlamydial protease-like activity factor
CXCL1: Chemokine C-X-C motif ligand 1
DEAE: Diethylaminoethyl
DMEM: Dulbecco’s Modified Eagle Medium
EB: Elementary body
EFNA1: Ephrin A1
FBS: Fetal bovine serum
GM-CSF: Granulocyte-macrophage colony-stimulating factor
GNG12: Protein subunit gamma 12
HBSS: Hank’s Balanced Salt Solution
IL: Interleukin
IMAC: Immobilized metal affinity chromatography
IPTG: Isopropyl β-D-1-thiogalactopyranoside
IRAK2: Interleukin-1 receptor-associated kinase 2
KDM6A: Lysine-specific demethylase 6A
LPS: Lipopolysaccharide
MALDI-TOF/TOF: Matrix-assisted laser desorption/ionization-tandem time of flight
MOMP: Major outer membrane
MS/MS: Tandem mass spectrometry
ORF: Open reading frames
PBMC: Peripheral blood mononuclear cells
PML: Promyelocytic leukemia
PVDF: Polyvinylidene difluoride
qRT-PCR: Quantitative Real-time polymerase chain reaction
SAT3: Streptothricin-acetyltransferase
SD: Standard deviation
SDS-PAGE: Sodium dodecyl sulfate–polyacrylamide gel electrophoresis
SPG: Sucrose phosphate glutamate buffer
TNFAIP3: Tumor necrosis factor alpha-induced protein 3
TNFα: Tumor necrosis factor alpha
