# Supplementary figures and images for: Chlamydia trachomatis plasmid-encoding Pgp3 protein induces secretion of distinct inflammatory signatures from HeLa cervical epithelial cells
Source: BMC Microbiol. 2023 Mar 4;23:58. doi: 10.1186/s12866-023-02802-3 (PMC9985209; doi:10.1186/s12866-023-02802-3)

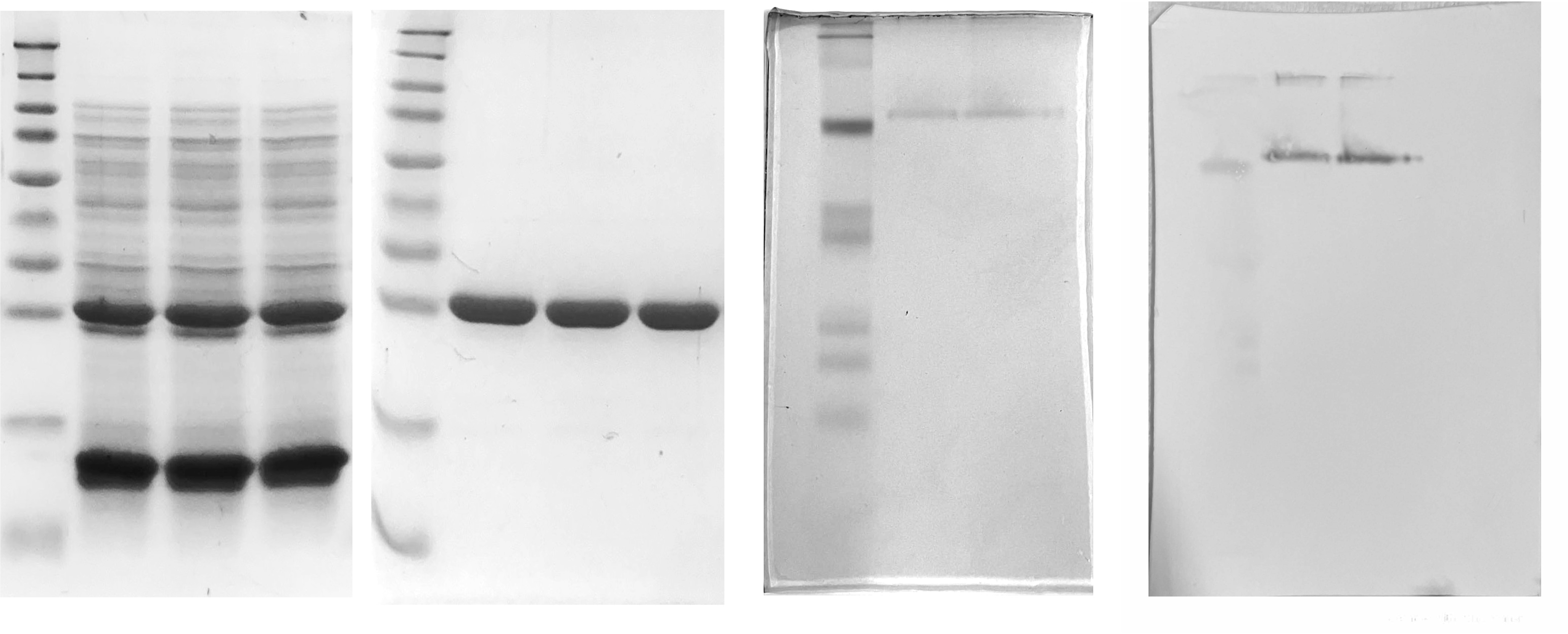

Supplement: Supplementary file 1 — Additional file 1. [file 12866_2023_2802_MOESM1_ESM.jpg]
